# Role of Non-Invasive Respiratory Supports in COVID-19 Acute Respiratory Failure Patients with Do Not Intubate Orders

**DOI:** 10.3390/jcm10132783

**Published:** 2021-06-24

**Authors:** Clément Medrinal, Alexis Gillet, Fairuz Boujibar, Jonathan Dugernier, Marcel Zwahlen, Bouchra Lamia, Christophe Girault, Jacques Creteur, Jean-Marc Fellrath, Laurence Haesler, Laurie Lagache, Laure Goubert, Elise Artaud Macari, Olivier Taton, Philippe Gouin, Dimitri Leduc, Olivier Van Hove, Michelle Norrenberg, Guillaume Prieur, Yann Combret, Nils Correvon, Roger Hilfiker, Olivier Contal

**Affiliations:** 1 UVSQ, Erphan, Paris-Saclay University, 78000 Versailles, France; 2 Intensive Care Unit Department, Le Havre Hospital, Avenue Pierre Mendes France, 76290 Montivilliers, France; laurie.lagache@ch-havre.fr; 3 Saint Michel School of Physiotherapy, Paris-Saclay University, 75015 Paris, France; 4 Department of Physiotherapy, Erasme University Hospital, Université Libre de Bruxelles, 1070 Bruxelles, Belgium; Alexis.Gillet@erasme.ulb.ac.be (A.G.); olivier.van.hove@erasme.ulb.ac.be (O.V.H.); michelle.norrenberg@erasme.ulb.ac.be (M.N.); 5 Inserm U1096, Rouen University Hospital, 76000 Rouen, France; fairuz.boujibar@chu-rouen.fr; 6 Department of General and Thoracic Surgery, Rouen University Hospital, 76000 Rouen, France; 7 Physiotherapy Unit, RHNe Réseau Hospitalier Neuchâtelois, Pourtalès Hospital, 2000 Neuchatel, Switzerland; jonathan.dugernier@rhne.ch (J.D.); nils.correvon@hesav.ch (N.C.); 8 Institute of Social & Preventive Medicine, University of Bern, 3012 Bern, Switzerland; marcel.zwahlen@ispm.unibe.ch; 9 Institute for Research and Innovation in Biomedicine (IRIB), Normandie Univ, UNIROUEN, EA3830-GRHV, 76000 Rouen, France; bouchra.lamia@chu-rouen.fr (B.L.); christophe.girault@chu-rouen.fr (C.G.); laurence.haesler@rhne.ch (L.H.); Elise.Artaud-Macari@chu-rouen.fr (E.A.M.); gprieur.kine@gmail.com (G.P.); yann.combret@gmail.com (Y.C.); 10 Pulmonology Department, Le Havre Hospital, Avenue Pierre Mendes France, 76290 Montivilliers, France; laure.goubert@ch-havre.fr; 11 Pulmonology, Respiratory Department, Rouen University Hospital, 76000 Rouen, France; 12 Intensive Care Unit Department, Rouen University Hospital, 76000 Rouen, France; Philippe.gouin@chu-rouen.fr; 13 Department of Intensive Care, Erasme University Hospital, Université Libre de Bruxelles, 1070 Bruxelles, Belgium; jacques.creteur@erasme.ulb.ac.be; 14 Pulmonary Medicine Unit, RHNe Réseau Hospitalier Neuchâtelois, Pourtalès Hospital, 2000 Neuchatel, Switzerland; Jean-Marc.Fellrath@rhne.ch; 15 Intensive Care Unit, RHNe Réseau Hospitalier Neuchâtelois, Pourtalès Hospital, 2000 Neuchatel, Switzerland; 16 Pulmonary, Thoracic Oncology and Respiratory Intensive Care Department, Rouen University Hospital, 76000 Rouen, France; 17 Department of Pneumology, Erasme University Hospital, Université Libre de Bruxelles, 1070 Bruxelles, Belgium; olivier.taton@erasme.ulb.ac.be (O.T.); dimitri.leduc@erasme.ulb.ac.be (D.L.); 18 Research and Clinical Experimentation Institute (IREC), Pulmonology, ORL and Dermatology, Louvain Catholic University, 1200 Brussels, Belgium; 19 School of Health Sciences (HESAV), HES-SO University of Applied Sciences and Arts Western Switzerland, 1011 Lausanne, Switzerland; olivier.contal@hesav.ch; 20 School of Health Sciences, University of Applied Sciences and Arts Western Switzerland Valais (HES-SO Valais-Wallis), 3954 Leukerbad, Switzerland; roger.hilfiker@gmail.com

**Keywords:** COVID-19, CPAP, high flow nasal cannula, mechanical ventilation, oxygenation

## Abstract

The current gold-standard treatment for COVID-19-related hypoxemic respiratory failure is invasive mechanical ventilation. However, do not intubate orders (DNI), prevent the use of this treatment in some cases. The aim of this study was to evaluate if non-invasive ventilatory supports can provide a good therapeutic alternative to invasive ventilation in patients with severe COVID-19 infection and a DNI. Data were collected from four centres in three European countries. Patients with severe COVID-19 infection were included. We emulated a hypothetical target trial in which outcomes were compared in patients with a DNI order treated exclusively by non-invasive respiratory support with patients who could be intubated if necessary. We set up a propensity score and an inverse probability of treatment weighting to remove confounding by indication. Four-hundred patients were included: 270 were eligible for intubation and 130 had a DNI order. The adjusted risk ratio for death among patients eligible for intubation was 0.81 (95% CI 0.46 to 1.42). The median length of stay in acute care for survivors was similar between groups (18 (10–31) vs. (19 (13–23.5); *p* = 0.76). The use of non-invasive respiratory support is a good compromise for patients with severe COVID-19 and a do not intubate order.

## 1. Introduction

Hospitals around the world have had to reorganise their services and increase their capacity due to the influx of hundreds of thousands of patients with severe COVID-19 infection. Furthermore, the severe acute respiratory failure and hypoxia induced by the disease has led to a widespread need for mechanical ventilation by orotracheal intubation [1,2,3]. Although invasive mechanical ventilation is the current gold-standard treatment for these conditions [4], it has some potential harmful effects on the pulmonary system. Prolonged mechanical ventilation has been associated with severe muscle weakness in all patients [5,6], and a high mortality rate for older patients and those with a low PaO_2_/FiO_2_ ratio [2,7]. Another issue with mechanical ventilation is that the massive influx of patients caused a shortage of equipment and induced an ethical challenge leading to a need for alternative methods of oxygenation [8,9].

In the context of limited resources, older patients and those with comorbidities, who represent a considerable proportion of patients with severe COVID-19 infection admitted to hospital, are often considered ineligible for invasive ventilation and are issued with a do not intubate (DNI) order. Therefore, during this pandemic, the use of non-invasive respiratory supports has increased throughout the world. High flow nasal cannula (HFNC) is the first line treatment to increase FiO_2_ and avoid intubation in patients with hypoxia [10]. Continuous Positive Airway Pressure (CPAP) can be used as a first or second line treatment, if HFNC therapy is insufficient, and its use in intensive care units (ICU) [11,12,13] as well as on conventional wards [14,15] has greatly expanded. Several treatment algorithms have been developed to guide clinicians in the non-invasive management of patients with severe COVID-19 infection [16,17]. Although the use of CPAP in the management of patients with COVID-19 is highly feasible, the results are uncertain. Several studies have reported a high rate of failure (40 to 66%) of this treatment [18,19,20], and a high rate of mortality in patients ineligible for intubation [13,21]. However, existing data are purely descriptive, and no definitive conclusions can be drawn regarding the efficacy of HFNC and CPAP in patients with a DNI order due to the lack of a comparison with a gold standard. The aim of this study is to determine if non-invasive ventilatory supports is a sufficient therapeutic alternative in patients with severe COVID-19 infection and a DNI.

## 2. Method

We conducted a multicentre retrospective study of data from patients admitted to 4 hospitals (Le Havre Hospital, Rouen University Hospital, Erasme Hospital, Réseau Hospitalier Neuchâtelois (RHNE) Pourtlaès Hospital) situated in three different European countries (France, Belgium and Switzerland) between the 1 October 2020 and 15 December 2020. All participating hospitals obtained ethical approval according to their country’s legislation.

Consecutive patients with severe COVID-19 infection (confirmed by PCR) who fulfilled the following criteria were included: age > 18 years, hospitalised in ICU or an intermediate care unit (IU), with high oxygen dependency (High Flow Nasal Cannula (HFNC) or PaO_2_/FiO_2_ < 300 or SpO_2_ < 94% with a non-rebreather mask and an O_2_ flow of at least at 10 L/min). 

Exclusion criteria were as follows: Patient already using home HFNC for another reason, patient intubated by emergency services out of hospital or in the emergency department, use of CPAP or Bi-level non-invasive ventilation (NIV) for a reason another than acute respiratory failure due to COVID-19 infection, patient refusal of ventilatory support. 

### 2.1. Procedure

In the four participating centres, all patients with severe COVID-19 infection and hypoxia were hospitalised in ICU or IU depending on bed availability and whether they were eligible or not for intubation. Details of the procedures in each centre are provided in the Supplementary file. The decision not to intubate was collegial and was based on age, comorbidities and bed availability in ICU. Depending on the patient’s respiratory status and equipment availability, each hospital used either HFNC (set at 50 L/min with FiO_2_ titrated for SpO_2_ > 92%) and/or CPAP (set between 8 and 12 cmH20 with FiO_2_ titrated for SpO_2_ > 92%). Respiratory support was primarily provided to treat acute respiratory failure due to hypoxemia, however if an increase in the partial pressure of carbon dioxide occurred, some patients were treated with Bi-level NIV (NIV). For patients with a Do Not Intubate (DNI) order, CPAP or Bi-level NIV was the ceiling treatment.

Due to the retrospective design of this study, the indications for intubation were not standardized: intubation was performed according to the judgement of the attending clinician. Nonetheless, indications for intubation in our ICU department include hypoxemia associated with marked respiratory failure, an inability to ensure airway protection, shock, and a reduced level of consciousness.

### 2.2. Main Reasons for Non-Intubation

The reasons for non-intubation were as follows: Patients older than 85 years, patients older than 80 years with a low level of autonomy and/or severe comorbidity (Chronic respiratory disease, chronic heart failure, etc.) and/or living with a mild frailty (Clinical Frailty scale ≥ 5), patients older than 80 years in the case of low ICU capacity (last ICU bed), patients with several comorbidities or living with a severe frailty (Clinical Frailty scale ≥ 7), and patients who refused orotracheal intubation.

### 2.3. Data Collection

Study personnel at each site collected data by manual chart review and used a standardized case report form to enter data into a secure online database. Data were collected using REDCap, a secure, HIPAA-compliant, web-based application. Potentials errors were all flagged in real time using the REDCap Data Quality module. After collection, all data were again manually reviewed, and values that appeared incongruent or out of range were manually validated by confirming the accuracy of the data with the collaborator who entered it.

Demographic characteristics, body mass index (BMI), comorbidities, date of COVID-19 diagnosis (positive PCR), date of hospital admission, biological examinations on admission to ICU/IU (including PaO_2_/FiO_2_ ratio, C-reactive protein, D-dimer and Fibrinogen) and quick-SOFA score on admission to ICU/IU were recorded. 

We chose not to use the SOFA score because the patients in IU did not undergo daily blood samples. Therefore, to avoid a large number of missing data, we used the quick-SOFA score [22] which summarises the severity of the patient’s condition using three items: (respiratory rate > 22 c/min; Glasgow Coma Scale score < 15/15; systolic blood pressure < 100 mmHg). This assessment has been evaluated for use in patients with COVID-19 [23]. When it was not possible to collect FiO_2_ data for a patient, we used the conversion table described by the COVID-ICU group of the REVA network [2].

Finally, use and duration of HFNC, CPAP, Bi-level NIV, invasive mechanical ventilation, medication (antibiotherapy, corticosteroids, hydroxychloroquine and tocilizumab) and clinical outcomes (mortality, ICU/IU length of stay and discharge destination) were collected. We chose not to collect data for interventions after intubation (neuromuscular blockers, prone position, ECMO, and weaning strategy) because we considered that the indications were not different from usual practice. Patients were followed up until death or discharge from ICU or IU.

### 2.4. Target Trial Framework

We sought to emulate a hypothetical target trial by classifying patients into two groups: Group 1 was composed of patients with severe COVID-19 with a DNI order who were treated with HFNC and/or CPAP as a ceiling treatment, and Group 2 was composed of patients who remained eligible for invasive mechanical ventilation.

### 2.5. Inverse Probability of Treatment Weighting

Because patients were categorized into one of these two groups using medical criteria, we set up a propensity score for the probability of belonging to Group 1 and an inverse probability of treatment weighting to remove confounding by indication. For the propensity score, a logistic regression model was fitted with (i) the variables that informed the decision to classify patients into Group 1 or Group 2 (age, age-squared and severe comorbidities) or (ii) the variables that are potentially associated with mortality (PaO_2_/FiO_2_, BMI, BMI-squared, administration of corticosteroids, quick-SOFA score, hypertension, time from diagnosis to admission, HFNC at admission and CPAP (or bilevel-NIV) at admission). The choice of variables to include was made a priori to the data analysis as they were thought to be potentially associated with a clinician’s decision to classify patients in Group 1 or 2 and with survival.

The overlap of the distributions of the variables between Group 1 and Group 2 was checked. Because no patient aged 85 or older was allocated to the Group 2 which was eligible for intubation, we excluded patients aged 85 or older from the primary analysis.

The model’s predicted probabilities for classification into the Group 1 were used to calculate stabilized inverse probability weights [24], which were then used to weight each individual’s contribution to the logistic regression for death. To account for the inverse probability weighting, we used a robust variance estimator.

### 2.6. Statistical Analysis

For missing data, we imputed ten data sets with multiple imputations and combined the results using Rubin’s rule [25]. Descriptive statistics were performed on non-imputed data, as recommended. 

We evaluated standardized differences across each measured covariate before and after applying the weighting. The balance of prognostic variables pre- and post-inverse probability of treatment weighting was evaluated with Cohen’s d for continuous variables and with odds ratios for binary variables. For continuous outcomes, we performed unweighted and weighted linear regressions, for binary outcomes we used logistic regression, and we calculated the Cohen’s d on unweighted (i.e., before inverse probability of treatment weighting) and weighted results (i.e., after inverse probability of treatment weighting).

We chose to report the overall mortality rate rather than time to death because invasive mechanical ventilation could increase the duration of survival, even in patients with a very low probability of surviving.

## 3. Results

**Figure 1:**
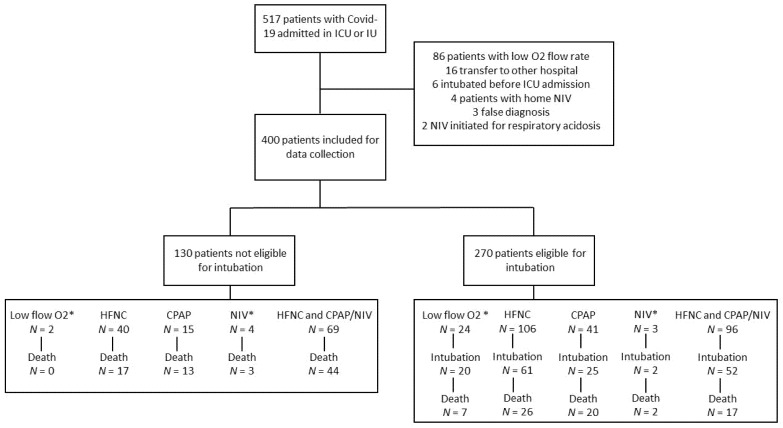
Flow chart. * Low flow O_2_ is defined by non-rebreather mask with O_2_ flow at least at 10 L/min. NIV: Bi-level NIV.

During the period studied, 517 patients were admitted to ICU or IU due to severe COVID-19 infection. Of these, 86 were sufficiently oxygenated with a low O_2_ flow rate, and a further 31 did not fulfil the inclusion criteria (See Figure 1). A total of 400 patients was included in the analysis.

Table 1 describes the patient characteristics and characteristics by centre are shown in Supplementary file (Table S1). Briefly, 130 (32.5%) were issued with a DNI order (Group 1) and 270 patients were classified as eligible for intubation and invasive mechanical ventilation (67.5%) (Group 2). In Group 1, 109 (84%) patients were treated with HFNC and 74 (57%) with CPAP therapy. In Group 2, 202 (75%) received HFNC and 118 (44%) received CPAP therapy. Finally, 161 (60%) had to be intubated. The median time from admission to intubation was 3 (2–6.5) days. Patients in Group 2 were younger (66 (58–73) vs. 79.5 (72–84) years; *p* < 0.0001) and had fewer comorbidities. There were no significant between group differences for the Quick-SOFA score, PaO_2_/FiO_2_ and C-reactive protein at admission. The durations of HFNC and CPAP therapy were significantly longer for patients in Group 1 (respectively., 6 (4–9) vs. 3 (2–5) days; *p* < 0.0001 and 6 (3–9) vs. 3 (2–7) days; *p* < 0.001).

**Figure 2:**
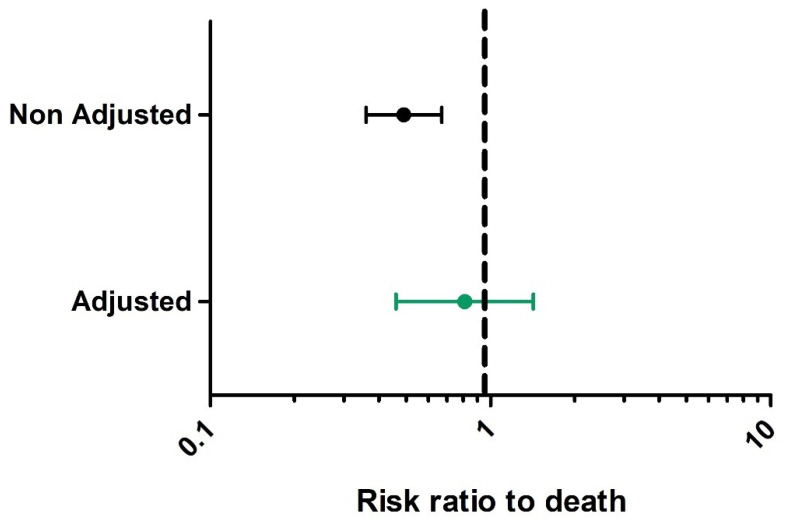
Black caterpillar plot represents overall risk ratio to death before inverse probability of treatment weighting. Green caterpillar plot represents risk ratio to death after inverse probability of treatment weighting.

In Group 2, 72 (27%) patients died compared to 77 (59%) in Group 1: risk ratio 0.49 (0.36–0.67). Once confounding factors were taken into account, the adjusted risk ratio for death among patients eligible for intubation was 0.81 (95% CI 0.46 to 1.42) (Figure 2).

**Figure 3:**
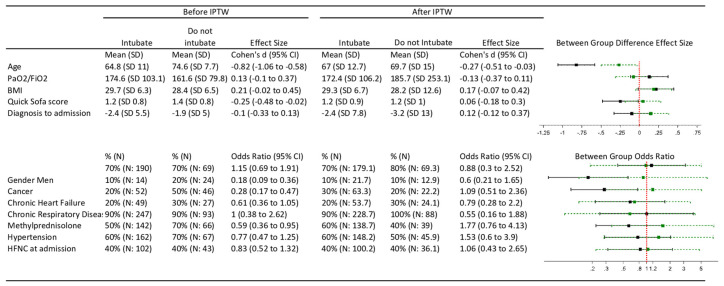
Characteristics of the patients before and after applying inverse probability of treatment weighting. Black Caterpillar plot represents risk ratio to death before inverse probability of treatment weighting. Green caterpillar plot represents risk ratio to death after inverse probability of treatment weighting. BMI: Body Mass Index; HFNC: High Flow Nasal Cannula; IPTW: Inverse Probability of Treatment Weighting; *n*: Number of patients; SD: Standardized Deviation.

Figure 3 shows the characteristics of the patients in both groups, before and after applying inverse probability of treatment weighting. The effect sizes for the differences between the two groups in the important prognostic factors were strongly reduced when the inverse probability of treatment weighting was applied. However, there was still a small difference for age: patients in Group 1 were older.

Factors associated with mortality are presented in Supplementary file (Figure S1). Risk ratio to death were higher for Older age, Chronic Heart Failure, Cancer and Neurological pathology comorbidities (respectively, 1.05 (1.03 to 1.06); 1.59 (1.14 to 2.21); 1.68 (1.09 to 2.6) and 2.34 (1.5 to 3.64)). Use of HFNC at admission and PaO_2_/FiO_2_ were associated with lower mortality (respectively, 0.62 (0.45 to 0.86) and 0.90 (0.82 to 0.99)).

**Figure 4:**
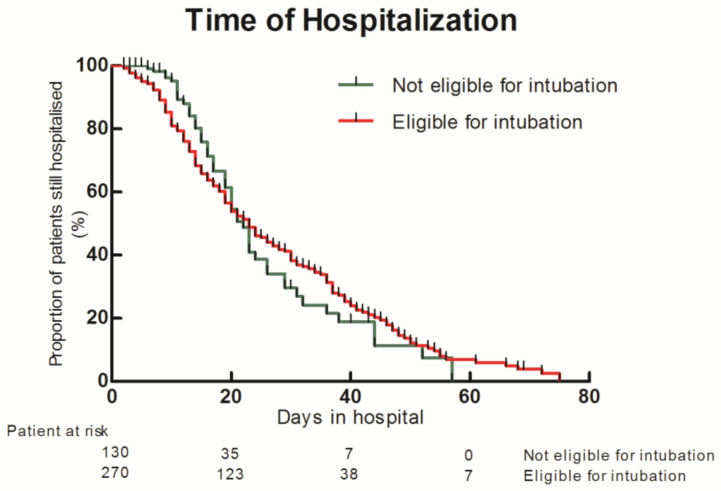
Kaplan–Meier of hospital length of stay between patient eligible to intubation and patients not eligible to intubation.

The median length of stay in ICU or IU was similar between groups for survivors (19 days (13–23.5) vs. 18 days (10–31); *p* = 0.76)) (Figure 4).

In Group 1, 16% of survivors were discharged home compared with 44% in Group 2. The proportion of patients discharged to a rehabilitation centre was similar between the two groups (respectively, 20% and 21%) (See Supplementary file: Figure S2).

## 4. Discussion

This is the first European-wide study to compare outcomes in patients with severe COVID-19 infection and a do not intubate order treated with non-invasive respiratory support to those patients eligible for treatment with invasive mechanical ventilation. Since a randomised, controlled trial would be unethical to conduct in such a situation, we simulated an intention to treat analysis in which patients received treatment according to the severity of their condition (and its progression), with adjustment by the covariables associated with mortality. The results suggest that (1) there was no significant difference in adjusted mortality rate between the patients with a DNI and those eligible for intubation, and (2) the duration of hospitalization was similar between survivors with a DNI order and those eligible for intubation.

The decision not to intubate patients with severe COVID-19 infection is not straightforward. It depends on the number of available beds and patient-related factors [8,9]. Until now, the high mortality rates among patients with a DNI order who were managed using non-invasive ventilatory supports, created uncertainty as to the benefits of such supports [15,21]: the results of this study provide some clarity regarding this issue. Although the unadjusted rate of mortality was higher for the patients with a DNI order, adjustment for the different mortality-related variables showed that the mortality rate was not statistically different from that of the patients eligible for intubation. These results suggest that for those patients with several comorbidities, invasive mechanical ventilation would not lead to better outcomes. 

The gold-standard treatment for respiratory failure in those who are eligible remains invasive mechanical ventilation. This treatment increases the short-term probability of survival and intubation provides a bridge for other rescue treatments, such as protective ventilation with alveolar recruitment, prone position [26] or extra corporeal membrane oxygenation [27]. Nevertheless, the efficacy of mechanical ventilation in patients with COVID-19 and severe ARDS is reduced [2], and long-term mortality is increased [28]. Furthermore, our results do not suggest a large benefit from invasive ventilation for patients with a DNI order. Indeed, a recent meta-analysis reported that the case fatality rate in patients with COVID-19 over the age of 60 years who are intubated is more than 70%, and increases exponentially with age, up to 84.4% in those above the age of 80 years [7]. Furthermore, in fragile and older patients, non-invasive respiratory support has the advantage of not requiring sedation and immobility. As such, transfers to the chair and rehabilitation can begin early, maintaining patient autonomy and reducing the physical and psychological consequences of mechanical ventilation [29,30]. Unfortunately, we were unable to report functional status at discharge from ICU/IU or hospital discharge, however the length of stay in ICU/IU was similar between groups, and the same proportion (20%) of patients were discharged to a rehabilitation centre.

A large proportion (78%) of the patients in this multicentre cohort were treated with HFNC at ICU/IU admission. The use of HFNC as a first line treatment for COVID-19-induced respiratory failure was proposed by Demoule et al. and appears to decrease the need for intubation at day 28 [10]. Despite this, HFNC failed to sufficiently reverse hypoxia in 56% of the patients in that study who were subsequently treated with invasive mechanical ventilation. We propose that in patients with a DNI order, CPAP could be a valid rescue option. Compared to HFNC, CPAP has the advantage of providing a high FiO_2_ at higher pressures, thus increasing alveolar oxygenation and decreasing respiratory muscle work [31]. However, these devices have also been reported to be potentially dangerous due to the generation of high tidal volumes and excessive transpulmonary pressure swings which can induce volutrauma [32] and the risk of delayed intubation [33]. The results of the present study showed that the rate of CPAP use was lower in patients eligible for intubation, probably due to the rapid use of invasive ventilation after HFNC failure. One of the major problems with CPAP is that it cannot be used continuously for several days because, even when optimally applied, the interface can be uncomfortable. In the present cohort, all types of interfaces were used (helmet, face mask and nasal pillows) depending on the habits of each department, equipment availability and patient toleration.

This study has several limitations. Firstly, it was not a randomized control trial and, even after adjustment, there was still a small difference in age between groups. However, our results are based on real life practices in ICUs and IUs where personalized, not protocolized, respiratory support is provided to patients. Secondly, the sample size was limited by the inclusion of only patients from the second wave of the pandemic in Europe (winter 2020). However, the advantage of this is that, by this time patients were treated with the highest level of evidence-based knowledge of the disease (e.g., 93% were treated with corticosteroids). The health-care teams were also experienced in the management of the disease, thus similar treatment protocols were applied across the four participating hospitals. Thirdly, published studies on that topic focused on CPAP or high flow nasal cannula alone, whereas both supports are very frequently combined in our centres, which is thought to provide better outcomes [34], and the superiority of those devices on one another has not yet been well established.

## 5. Conclusions

This comparison of the outcomes of treatment with non-invasive respiratory support in patient’s ineligible and eligible for intubation found that the use of non-invasive respiratory support is a good compromise for patients with severe COVID-19 and a do not intubate order. Future research is required to further define the modalities of application of non-invasive ventilatory supports.

## Figures and Tables

**Table 1:** Population characteristics.

Characteristics	All Population	Do Not Intubate Order (*n* = 130)	Intubation Eligible (*n* = 270)	*p* Value
Male, *n* (%)	284 (71)	87 (67)	197 (73)	0.21
Age, years	70 (61.2–78)	79.5 (72–84)	66 (58–73)	<0.0001
Body Mass Index, kg/m^2^	28.4 (24.8–32)	27.1 (23.2–31.1)	29 (25.2–32.4)	0.003
Delay between PCR diagnostic and admission, days	0 (0–5)	1 (0–5)	0 (0–5)	0.51
Comorbidities				
Chronic respiratory disease, *n* (%)	81 (20)	32 (24)	49 (18)	0.13
Chronic heart disease, *n* (%)	110 (27)	58 (45)	52 (19)	<0.0001
Hypertension, *n* (%)	228 (57)	83 (64)	145 (54)	0.055
Diabetes, *n* (%)	175 (44)	55 (42)	120 (45)	0.68
Obesity, *n* (%)	149 (37)	40 (31)	109 (40)	0.06
Cancer, *n* (%)	41 (10)	27 (21)	14 (5)	<0.0001
Neurological pathology, *n* (%)	29 (7)	19 (15)	10 (3)	<0.0001
Cognitive disorders, *n* (%)	37 (9)	18 (14)	19 (7)	0.03
At admission				
Quick SOFA total score	1 (1–2)	1 (1–2)	1 (1–2)	0.09
Respiratory rate > 22 c/min, *n* (%)	332 (83)	107 (83)	225 (83)	0.79
Glasgow score < 15, *n* (%)	80 (20)	40 (31)	40 (15)	0.0002
Systolic BP < 100 mmHg, *n* (%)	71 (18)	21 (16)	50 (18)	0.56
PaO_2_/FiO_2_, (mmHg)	140 (95–225)	138 (101–205)	143 (92–234)	0.6
C-reactive protein, (mg/L)	113 (62.8–170)	115 (76–140)	111.5 (53.7–180.3)	0.75
D-dimer, (µg/L)	1193 (731–2267)	1387 (953–2923)	1098 (700–2000)	0.019
Fibrinogen, (g/L)	6.7 (5.8–7.5)	6.4 (5.2–7.4)	6.8 (5.9–7.6)	0.11
Respiratory Support				
HFNC, *n* (%)	311 (78)	109 (84)	202 (75)	0.04
Delay between HFNC and Admission, days	1 (0–4)	2 (0–4)	1 (0–4)	0.37
HFNC duration, days	4 (2–7)	6 (4–9)	3 (2–5)	<0.0001
CPAP, *n* (%)	192 (48)	74 (57)	118 (44)	0.013
Delay between CPAP and Admission, days	2 (0–4)	3 (1–5)	1 (0–4)	0.01
CPAP duration, days	4 (2–8)	6 (3–9)	3 (2–7)	0.001
Bi-level NIV, *n* (%)	51 (13)	20 (15)	31 (12)	0.27
Delay between Bi-level NIV and Admission, days	2.5 (0–7)	8 (0–14)	2 (0–5)	0.025
Bi-level NIVduration, days	3 (2–5)	3.5 (2–6)	2 (1–3	0.1
IMV, *n* (%)	161 (60)	-	161 (60)	-
Delay between IMV and Admission, days	3 (2–6.5)	-	3 (2–6.5)	-
IMV duration, days	13 (8–24)	-	13 (8–24)	-
Medication				
Remdesivir, *n* (%)	41 (10)	18 (14)	23 (9)	0.09
Plaquenil, *n* (%)	1 (0.2)	0 (0)	1 (0.3)	0.48
Tozicilumab, *n* (%)	26 (6)	9 (7)	17 (6)	0.81
Corticosteroids, *n* (%)	374 (93)	120 (92)	254 (94)	0.5
Antibiotherapy, *n* (%)	290 (72)	87 (67)	203 (75)	0.08
Meropenem, *n* (%)	56 (14)	9 (7)	47 (18)	0.005

PCR: Polymerase Chain Reaction; Systolic BP: Systolic blood pressure; HFNC: High Flow Nasal Cannula; CPAP: Continuous Positive Airway Pressure; NIV: Non-invasive ventilation IMV: Invasive Mechanical Ventilation.

## Data Availability

Clément Medrinal had full access to all the data in the study and takes responsibility for the integrity of the data and the accuracy of the data analysis. Data can be obtained from the corresponding author: Clément Medrinal; medrinal.clement.mk@gmail.com.

## Supplementary Materials

The following are available online at https://www.mdpi.com/article/10.3390/jcm10132783/s1, Supplementary file of Role of non-invasive respiratory supports in COVID-19 acute respiratory failure patients with do not intubate order, Table S1. Population characteristics by centre, Figure S1. Risk ratio of mortality according to unadjusted variable, Figure S2. Orientation after Hospital discharge.
