# Supplementary material for: Role of Non-Invasive Respiratory Supports in COVID-19 Acute Respiratory Failure Patients with Do Not Intubate Orders
Source: J Clin Med. 2021 Jun 24;10(13):2783. doi: 10.3390/jcm10132783 (PMC8267931; doi:10.3390/jcm10132783)
Supplement: Supplementary file 1 [file jcm-10-02783-s001.zip › jcm-1264736-supplementary.pdf]

Role of non-invasive respiratory supports in Covid-19 acute respiratory failure patients with do not intubate order.

Clément Medrinal, Alexis Gillet, Fairuz Boujibar, Jonathan Dugernier, Marcel Zwahlen, Bouchra Lamia, Christophe Girault, Jacques Creteur, Jean Marc Fellrath, Laurence Haesler, Laurie Lagache, Laure Goubert, Elise Artaud Macari, Olivier Taton, Philippe Gouin, Dimitri Leduc, Olivier Van Hove, Michelle Norrenberg, Guillaume Prieur, Yann Combret, Nils Correvon, Roger Hilfiker, Olivier Contal

Supplemental Method

Centre Organisation

Le Havre Hospital, France

The dedicated Covid-19 ICU was composed of 27 beds. A 25-bed intermediate unit was created for patients who required an O_2 flow rate above 6L/min to maintain SpO_2 > 92%, patients waiting for a place in ICU and those with a DNI order.

HFNC set at 50L/min was the first line respiratory therapy for all patients and CPAP could be used in patients with a DNI order if HFNC was not sufficient. CPAP therapy was provided with ICU or home ventilators via face masks or nasal pillows.

Rouen University Hospital, France

The dedicated Covid-19 ICU was composed of 42 beds for both patients eligible for invasive ventilation and those with a DNI order. Patients were admitted to ICU if they required an O_2 flow above 5L/min to maintain SpO_2 > 92%. There was no IU. HFNC set at 50L/min was the first line respiratory therapy for all patients and CPAP could be used in patients with a DNI

order if HFNC was not sufficient. CPAP therapy was provided with ICU or home ventilators via face masks.

Erasme Brussels, Belgium

The ICU contained 20 beds for patients with Covid-19. A 12-bed IU was created for patients who required an O_2 flow above 6L/min to maintain SpO_2 > 92%, patients waiting for a place in ICU and those with a DNI order. CPAP was the first line therapy because of a lack of availability of HFNC. CPAP therapy was administered using a pneumatic device via face masks or helmets.

Pourtalès Hospital, Switzerland

The ICU contained 13 beds for patients with Covid-19. A 10-bed IU was created for patients who required an O_2 flow above 6L/min to maintain SpO_2 > 92%, patients waiting for a place in ICU and those with a DNI order. HFNC set at 50L/min was the first line respiratory therapy for all patients and CPAP could be added if HFNC was not sufficient. CPAP therapy was administered with ICU or home ventilators via facial masks.

Results

Table S1. Population characteristics by centre.

Characteristics	Le Havre Hospital France	Rouen University Hospital France	Erasme Hospital Belgium	RHNE Switzerland
Male, n (%)	71 (66)	86 (75)	74 (73)	53 (78)
Age, years	74 (66-82)	67.5 (59-75)	65 (58.5-73.5)	74 (66.5-80)
Body Mass Index, Kg/m²	29.4 (26.2-33.1)	28.5 (24.8-32.9)	27.2 (24.5-30.9)	28.7 (23.6-31.1)
Delay between PCR diagnostic and admission, days	2.5 (0-6)	0 (0-5)	1 (0-4)	0 (0-4)
Comobidities				
Chronic respiratory disease, n (%)	25 (23)	12 (10)	24 (24)	20 (26)
Chronic heart disease, n (%)	36 (33)	29 (25)	17 (17)	28 (36)
Hypertension, n (%)	59 (55)	69 (60)	57 (56)	43 (56)
Diabetes, n (%)	42 (39)	59 (52)	44 (43)	30 (39)
Obesity, n (%)	49 (45)	44 (39)	30 (30)	26 (34)
Cancer, n (%)	11 (10)	16 (14)	8 (8)	6 (8)
Neurological pathology, n (%)	12 (11)	3 (3)	5 (5)	9 (12)
Cognitive disorders, n (%)	6 (6)	9 (8)	8 (8)	14 (18)
At admission				
Quick SOFA total score	1 (0.25-1)	2 (1-2)	1 (1-1)	1 (1-1)
Respiratory rate >22c/min, n (%)	77 (71)	111 (97)	84 (83)	60 (78)
Glasgow score < 15, n (%)	13 (12)	35 (31)	22 (22)	10 (13)
Systolic BP < 100 mmHg, n (%)	7 (6)	54 (47)	5 (5)	5 (6)
PaO_2/FiO_2, (mmHg)	105 (71-143)	179 (106-242)	160 (104-280)	158 (86-233)
C-reactive protein, (mg/L)	117 (78-173)	92 (34-176)	120 (81-170)	110 (62-156)
D-dimer, (µg/L)	1457 (921-2716)	1089 (728-1912)	953 (553-227)	1255 (828-1933)
Fibrinogen, (g/L)	6.7 (5.4-7.6)	6.7 (6.1-7.3)	Not collected	4.3 (3.2-6.6)
Respiratory Support				
HFNC, n (%)	100 (93)	101 (89)	42 (42)	68 (88)
Delay between HFNC and Admission, days	1 (0-3)	1 (0-3)	1 (0-3)	2 (1-5)
HFNC duration, days	4 (2-8))	4 (2-7)	3 (2-5)	4 (2-8)
CPAP, n (%)	29 (27)	29 (26)	90 (89)	44 (58)
Delay between CPAP and Admission, days	3 (1.5-6)	3 (1-4.5)	1 (0-3)	3 (1-6)
CPAP duration, days	5 (2-9)	4 (2-10.5)	4 (2.5-7.5)	4 (2-8)
Bi-level NIV, n (%)	9 (8)	17 (15)	6 (6)	19 (24)
Delay between Bi-level NIV and Admission, days	4.5 (0-11)	2 (0-7.5)	3.5 (0-10	3 (1-6)
Bi-level NIV duration, days	6 (1.5-10)	3 (2-5.5)	1.5 (1-3.5)	2 (2-4)
IMV, n (% overall group)	37 (34)	47 (41)	40 (40)	37 (48)
IMV, n (% Intubable)	37 (64)	47 (59)	40 (51)	37 (68)
Delay between IMV and Admission, days	2 (1-7.5)	3 (1-5)	5 (3-10)	3 (2-6)
IMV duration, days	12 (8-27)	19 (11-27)	12.5 (6.5-22)	10 (6-16.5)
Medication				
Remdesivir, n (%)	6 (6)	25 (22)	8 (8)	2 (3)
Plaquenil, n (%)	0 (0)	0 (0)	1 (1)	0 (0)
Tozicilumab, n (%)	1 (0.9)	1 (0.8)	6 (6)	18 (23)
Corticosteroids, n (%)	101 (94)	99 (87)	100 (99)	74 (96)
Antibiotherapy, n (%)	92 (85)	70 (61)	59 (58)	74 (96)
Meropenem, n (%)	0 (0)	20 (17)	14 (15)	22 (28)

Systolic BP : Systolic blood pressure ; HFNC : High Flow Nasal Cannula ; CPAP : Continuous Positive Airway Pressure ; IMV : Invasive Mechanical Ventilation

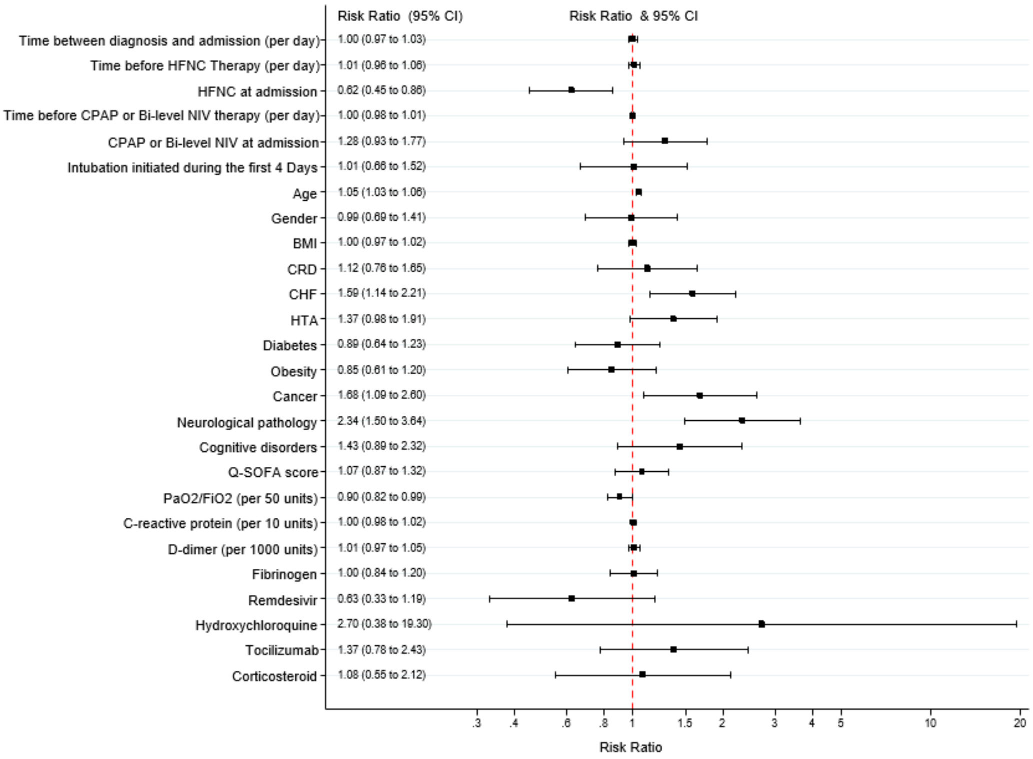

Figure S1. Risk ratio of mortality according to unadjusted variable. HFNC: High Flow Nasal Cannula; CPAP: Continuous Positive Airway Pressure; BMI: Body Mass Index; CRD: Chronic Respiratory Disease; CHF: Chronic Heart Failure; HTA: Hypertension; Q-SOFA: Quick SOFA

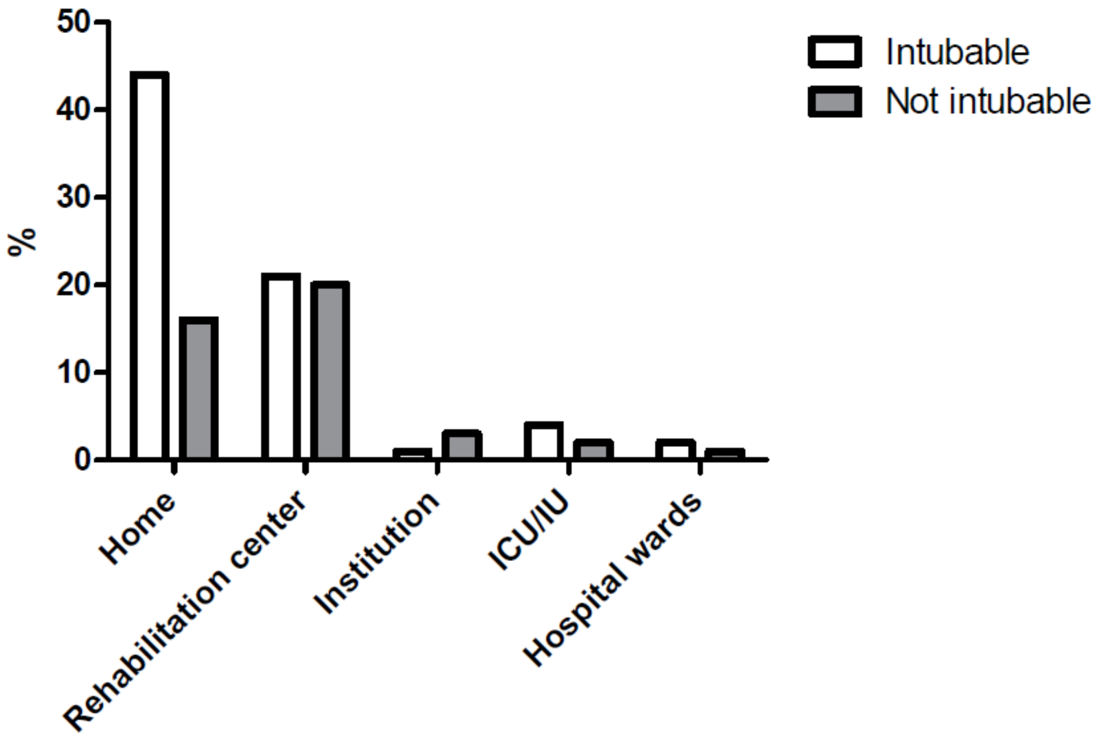

Figure S2. Orientation after Hospital discharge.
